# Relationship between vascular endothelial growth factor gene polymorphisms and Crimean–Congo hemorrhagic fever

**DOI:** 10.1590/1806-9282.20250147

**Published:** 2025-08-08

**Authors:** Umut Safiye Şay Coşkun, Serbülent Yiğit, Recai Aci, Ercan Tural, Nihan Bozkurt, Hüseyin Şener Barut

**Affiliations:** 1 Tokat Gaziosmanpaşa University, Faculty of Medicine, Department of Medical Microbiology – Tokat, Türkiye.; 2 Ondokuz Mayıs University, Faculty of Veterinary Medicine, Department of Genetics – Samsun, Türkiye.; 3 Aydın Adnan Menderes University, Söke Vocational School of Health Services – Aydın, Türkiye.; 4 Ondokuz Mayıs University, Faculty of Health Sciences, Department of Physiotherapy and Rehabilitation, Division of Physiotherapy and Rehabilitation – Samsun, Türkiye.; 5 Tokat Gaziosmanpaşa University, Faculty of Medicine, Department of Medical Biology – Tokat, Türkiye.; 6 Ministry of Health, Tepecik Training and Research Hospital, Department of Infectious Disease – İzmir, Türkiye.

**Keywords:** Crimean–Congo hemorrhagic fever, Vascular endothelial growth factor, Gene polymorphism, Vascular endothelial growth factor A/genetics, Single nucleotide polymorphism

## Abstract

**OBJECTIVE:**

This study aims to investigate the relationship between vascular endothelial growth factor gene polymorphisms and Crimean–Congo hemorrhagic fever

**METHODS:**

A total of 130 serum samples were analyzed, including 60 patients diagnosed with Crimean–Congo hemorrhagic fever and 70 healthy controls. Genetic analyses were performed using polymerase chain reaction and restriction fragment length polymorphism techniques.

**RESULTS:**

Significant differences were observed between patient and control groups in terms of vascular endothelial growth factor −460 C/T and -ID (18 bp) polymorphisms.

**CONCLUSION:**

Vascular endothelial growth factor gene polymorphisms may contribute to genetic susceptibility to Crimean–Congo hemorrhagic fever. Further studies with larger sample sizes are recommended to support these findings.

## INTRODUCTION

Crimean–Congo hemorrhagic fever (CCHF) is a potentially lethal zoonotic disease caused by a tick-borne arbovirus. CCHF Orthonairovirus, a negative-polarity, single-stranded RNA virus, is the causal agent^1^. After exposure to the virus, following an incubation period of 3–7 days, in more severe clinical forms, skin and mucosal hemorrhages are added to this picture, and a fatal course ranging from 5 to 30% is observed in the disease^2^. The endothelium is thought to be a significant target in CCHF. Hemorrhage, increased vascular permeability, and thrombocytopenia are important markers leading to the clinical exacerbation of the disease^3^.

Vascular endothelial growth factor-A (VEGF-A) is an important factor involved in both inflammation and plasma leakage in CCHF disease^4^. The VEGF signaling pathway is considered to be rate-limiting in physiological angiogenesis. The VEGF family consists of seven glycoproteins^5^.

The promoter region is an important part of the gene that regulates its expression. The VEGF polymorphism gene may have an important effect on the development of a variety of medical problems with inflammatory processes, such as psoriasis, rheumatoid arthritis, and colon cancer^6-8^. It is crucial to highlight, however, that the exact mechanisms and significance of this polymorphism in the development of various disorders are still being researched.

This study aimed to investigate whether the insertion/deletion (I/D) polymorphism of the 18 bp fragment at position −2549 of the VEGF −460 C/T and VEGF gene promoter regions is associated with CCHF disease.

## METHODS

### Study samples

We collected a total of 130 samples from 60 patients (22 females, 38 males; mean age: 37.48/60.81 years) diagnosed with CCHF and 70 healthy controls (32 females, 38 males; mean age: 43.78/53.76 years) for DNA isolation. These samples were obtained from a blood sample, which was collected in an ethylenediaminetetraacetic acid (EDTA) tube.

### Genotype analysis

Detection of I/D polymorphism (rs35569394) of the VEGF at the −2549 position in the promoter region was performed with the use of the "5’ GCTGAGGATGGGGCTGACTAGGTA 3’" and "5’ GTTT CTGACCTGGCTATTTCCAGG 3’" primers. In each reaction, we used 50 ng of DNA along with one concentration of each primer, 10 μM concentrations of each dNTP, 1.5 mM MgCl_2_, 0.2 units of Taq polymerase, and 10x polymerase chain reaction (PCR) buffer in a volume of 25 μl. To visualize the amplified PCR products, we used a gel made from a solution containing agarose with a concentration of 2.5%.

To perform the analysis, we collected DNA from blood using the Extractor WB kit manufactured by Wako, Japan. We then performed PCR on the target genes. Each PCR reaction was prepared in a 50-μL volume containing DNA, 2.6 pmol of each primer, 1X Taq polymerase buffer (containing 1.5 mM MgCl_2_), and 0.25 units of AmpliTaq DNA polymerase manufactured by Perkin Elmer in Foster City, CA. For the VEGF gene −460 C/T BstUI polymorphism, the specific primers used for PCRs were as follows: 5’ TGTGCGTGTGGGGTGAGCG 3’ and 5’ TACGT GCGGACACAGGGCCTGA 3’. PCR amplification was performed using a cycler called GeneAmp PCR System 2400, manufactured by Perkin Elmer. The resulting PCR product was 175 base pairs (bps) in length. We followed the instructions provided by the manufacturer. The BstU I restriction site was located exactly at the "C to T" transition at bp 460 of exon I. After incubating the digestion products for 2 h at 37°C, we loaded 10 μL of these products onto a 3% agarose gel for electrophoresis. We divided the polymorphism into three genotypes: "TT" (homozygous), "CC" (non-cuttable homozygous), or "TC" (heterozygous).

### Statistical analysis

Statistical analysis was carried out using SPSS 20 (SPSS Inc., Chicago, IL, USA). The genotype distributions and allele frequencies were calculated using chi-square analysis. For genotype distributions as well as grouped genotype and allele comparisons, the OpenEpi program was used. The statistical analysis results acknowledged p>0.05 as statistically significant.

## RESULTS

### Genotype and allele frequencies of the vascular endothelial growth factor gene

In this study, the genotype distributions of VEGF −460 C/T and I/D variants of the 18 bp fragment at position −2549 of the promoter region in the VEGF gene were analyzed in patient and control groups. A total of 60 patients and 70 control samples were analyzed.

According to the findings regarding VEGF 460 T/C genotype distribution, the rate of individuals with the TT genotype was found to be 18.3% among the patients, while this rate was 24.3% in the control group. The rate of TC genotype was 55% among the patients, while this rate was 57.1% in the control group. The CC genotype was determined to be 26.7% in the patients and 18.6% in the control group. The differences between the genotype distributions were found to be statistically significant (χ^2^=132.519, p<0.001). In terms of allele distribution, the T allele was found in 46% of the patients and 52% in the control group. The C allele was 54% in patients and 48% in the control group. The differences between allele distributions were not statistically significant (χ^2^=1.032, p<0.311). Genotype and allele frequencies of the VEGF gene 460 T/C polymorphism are shown in Table 1.

**Table 1:** Clinical laboratory findings.

Laboratory findings	Patients (n=60) (mean±SD)	Min–max
Serum AST (U/L)	304.38±37.300	229.62–379.138
Serum ALP (U/L)	197.78±30.504	197.76–136.740
Serum PLT (U/L)	38.250±2.605	38.250–33.037
Serum aPTT (s)	49.020±1.793	49.015–45.430
Serum PT (s)	14.470±3.342	14.468–13.600

AST: alanine aminotransferase; PLT: platelet; aPTT: activated partial thromboplastin time; PT: prothrombin time; SD: standard deviation.

When the VEGF gene ID (18 bp) variant was analyzed, the genotype distributions analyzed in three different categories were classified as II, ID, and DD in the analyses performed on a total of 60 patients and 70 control samples in the same number and group of patients. According to the genotype distributions, it was observed that the rate of individuals with II genotype was 15% among the patients. In the control group, this rate was 17.1%. ID genotype was found in 46.7% of the patients, while this rate was 54.3% in the control group. DD genotype was found in 38.3% of the patients, while this rate was 28.6% in the control group. The differences between the genotype distributions were found to be statistically significant (p<0.001). The genotype and allele frequencies of the VEGF gene ID (18 bp) polymorphism are shown in Table 2.

**Table 2:** Genotype and allele frequencies of vascular endothelial growth factor gene 460 T/C polymorphism.

Genotype/allele	Patients (n=60)		Controls (n=70)		p-value
Genotype	n	%	n	%	
TT	11	18.3	17	24.3	p<0.001*
TC	33	55	40	57.1	
CC	16	26.7	13	18.6	
Total	60		70		
Allele	n	%	n	%	p-value
T	55	46	72	52	p<0.311
C	65	54	66	48	

* Statistically significant values are denoted in bold (p<0.05).

When the allele distributions were analyzed, the I allele was found in 38% of the patients and 44% in the control group. The D allele was 62% among the patients and 55% in the control group. The differences between the allele distributions were not statistically significant (p<0.135). The genotype and allele frequencies of the VEGF ID (18 bp) gene polymorphism are shown in Table 3.

**Table 3:** Genotype and allele frequencies of vascular endothelial growth factor gene ID (18 bp) polymorphism.

Genotype/allele	Patients (n=60)		Controls (n=70)		p-value
Genotype	n	%	n	%	
II	9	15	12	17.1	p<0.001*
ID	28	46.7	38	54.3	
DD	23	38.3	20	28.6	
Total	60		70		
Allele	n	%	n	%	p-value
I	46	38	62	44	p<0.135
D	74	62	78	55	

* Statistically significant values are denoted in bold (p<0.05).

## DISCUSSION

The CCHF virus primarily affects the endothelium and is associated with microvascular instability and impaired blood clotting. However, there is still an understanding regarding the pathogenesis of CCHF^9^.

The main proangiogenic factor in angiogenesis is VEGF. VEGF is a key factor in the development of new blood vessels, as well as in the regulation of blood vessel physiology during angiogenesis and inflammation^10^. VEGF is also a significant vascular permeability factor. Vascular dysfunction is an eminent component in the pathophysiology of CCHF, causing hemorrhages and death in the latter stages of the disease. VEGF serum levels were observed to be considerably greater in CCHF patients in investigations^11^.

Polymorphisms present in various genes can cause changes in gene expression, and there are several studies reporting that these patients are predisposed to CCHF disease^12,13^. We found an association between eNOS gene T786C polymorphisms and CCHF disease^14^. According to Engin et al., the frequency of homozygous mutant TLR3 genotypes in CCHF patients was substantially greater than that in healthy controls. Furthermore, the existence of the TLR3 (c.1377C/T and −7A/C) TT genotype may affect CCHF sensitivity^15^. According to Karakus et al., there is no difference in the allele and genotype distributions of the methylene-tetrahydrofolate reductase C677T and A1298C gene polymorphisms between CCHF patients and healthy controls. The frequency of the BT–AA composite genotype consisting of C677T–A1298C polymorphisms was determined to be substantially greater in moderate CCHF patients than in severe CCHF patients and the control group in the composite genotype comparison between various groups^13^. TLR7 gene variations in CCHF patients were also reported in another investigation. These gene variations may play a role in CCHF susceptibility or clinical course^16^.

Patients with polycystic ovarian syndrome exhibit comparable rates of VEGF polymorphisms rs2010963 and rs833061 as the general population^17^. According to the meta-analysis conducted in 2024, the VEGF −460 C/T gene polymorphism is related with vulnerability to diabetic retinopathy in Type 2 diabetes mellitus^18^. A study carried out in Brazil has shown that VEGF gene polymorphism may increase the potential for rapid tumor progression in people infected with hepatitis C. It has also been reported that patients diagnosed with hepatocellular cancers with polymorphisms are more likely to be diagnosed with multinodular disease and may have a negative impact on response to treatment^19^.

The clinical characteristics and outcomes of infectious diseases differ from patient to patient. Several ideas emphasize the significance of this heterogeneity. However, genetic variables influence susceptibility to infectious diseases^20^. To summarize, when we examine the distribution of both genetic variations between the patient and the control group, different genotype and allele distributions are observed between the two groups. In the first genetic variation, the TT and TC genotypes are more common among the patients, while in the second variation, the ID genotype is slightly higher in the control group. The differences in allele distributions were not statistically significant in both analyses, whereas the differences in genotype distributions were statistically significant in the first analysis. The limitation of this study is that our study group was not large.

In conclusion, these findings demonstrate that specific genetic variants may be linked to illness risk and etiology. However, larger sample size investigations and analyses are required to corroborate these findings. The VEGF gene 460 T/C and VEGF gene ID (18 bp) variants may be genetic markers for CCHF disease risk. To the best of our knowledge, this is the first study to show a link between VEGF gene 460 T/C and VEGF gene ID (18 bp) variant polymorphisms and CCHF illness.

## Data Availability

The datasets generated and/or analyzed during the current study are available from the corresponding author upon reasonable request.
